# Greenhouse gas emissions from dung pats vary with dung beetle species and with assemblage composition

**DOI:** 10.1371/journal.pone.0178077

**Published:** 2017-07-12

**Authors:** Irene Piccini, Fabrizio Arnieri, Enrico Caprio, Beatrice Nervo, Simone Pelissetti, Claudia Palestrini, Tomas Roslin, Antonio Rolando

**Affiliations:** 1 Department of Life Science and Systems Biology, University of Turin, Turin, Italy; 2 Department of Ecology, Swedish University of Agricultural Sciences, Uppsala, Sweden; 3 Department of Agricultural, Forest and Food Sciences, University of Turin, Grugliasco (TO), Italy; Universidade de São paulo, BRAZIL

## Abstract

Cattle farming is a major source of greenhouse gases (GHGs). Recent research suggests that GHG fluxes from dung pats could be affected by biotic interactions involving dung beetles. Whether and how these effects vary among beetle species and with assemblage composition is yet to be established. To examine the link between GHGs and different dung beetle species assemblages, we used a closed chamber system to measure fluxes of carbon dioxide (CO_2_), methane (CH_4_) and nitrous oxide (N_2_O) from cattle dung pats. Targeting a total of four dung beetle species (a pat-dwelling species, a roller of dung balls, a large and a small tunnelling species), we ran six experimental treatments (four monospecific and two mixed) and two controls (one with dung but without beetles, and one with neither dung nor beetles). In this setting, the overall presence of beetles significantly affected the gas fluxes, but different species contributed unequally to GHG emissions. When compared to the control with dung, we detected an overall reduction in the total cumulative CO_2_ flux from all treatments with beetles and a reduction in N_2_O flux from the treatments with the three most abundant dung beetle species. These reductions can be seen as beneficial ecosystem services. Nonetheless, we also observed a disservice provided by the large tunneler, *Copris lunaris*, which significantly increased the CH_4_ flux–an effect potentially traceable to the species’ nesting strategy involving the construction of large brood balls. When fluxes were summed into CO_2_-equivalents across individual GHG compounds, dung with beetles proved to emit less GHGs than did beetle-free dung, with the mix of the three most abundant species providing the highest reduction (-32%). As the mix of multiple species proved the most effective in reducing CO_2_-equivalents, the conservation of diverse assemblages of dung beetles emerges as a priority in agro-pastoral ecosystems.

## Introduction

Grazing animals release large amounts of nitrogen and carbon through their excreta in pastures. The excess of nutrients creates a release of Green House Gases (GHGs) which steadily leaks into the atmosphere [1,2]. The dung produced by livestock, in particular, is a significant source of GHGs such as nitrous oxide (N_2_O), methane (CH_4_), and carbon dioxide (CO_2_) [3–7]. GHG emissions from dung are primarily and directly dependent on microbiological processes. CO_2_ originates from the decomposition of organic material by micro-organisms, CH_4_ from methanogenic bacteria thriving in anoxic conditions and N_2_O from microbial nitrification, denitrification and nitrifier denitrification, i.e. nitrite reduction by ammonia oxidizers [8–13].

Yet, GHG fluxes are also affected by the macroscopic fauna. Recent studies reveal that dung beetles (Coleoptera: Scarabaeoidea) may influence the GHG emissions by aerating the dung and soil, by reducing organic matter, by relocating dung and by altering microbe communities [14–16]. Importantly, studies of beetle-mediated effects on GHG emissions have so far been focused on the general effect of either including or excluding dung beetles [17–19], or on the effects of single species [20]. In contrast, the effects of variation in species identity and community composition has received little or no attention. This status quo clashes with a general interest in the functional correlates of overall species diversity (from e.g. [21]), and of nesting strategies within species assemblages [22], with a general review in [23]. What these studies reveal is that even within larger assemblages, the level of ecosystem functioning may often be hinged on the presence of specific species [24]. Thus, to understand overall fluxes of GHGs from dung, we need to dissect the functional contributions of different dung beetle assemblages, and of individual taxa within such assemblages.

Importantly, different dung beetle taxa can be expected to modify gas fluxes to different extents. Dung beetle taxa vary in their nesting strategies, and can be divided in dwellers, tunnelers and rollers [25]. Of these, the ‘dwellers’ do not translocate dung but utilise dung pats by living inside them. The ‘tunnelers’ translocate dung to tunnels dug underneath the dung pat, whereas the ‘rollers’ first translocate pieces of dung horizontally, then bury them vertically. *A priori*, these different nesting strategies and/or the body mass of the species may significantly affect ecological function, such as dung removal efficiency [22,26–29]. As they result in *inter alia* holes of different diameter in different parts of the dung pat, and in different sizes of brood balls being translocated to different micro-environments, we hypothesized that they may also affect GHG fluxes differently. To test this hypothesis, we quantified GHG emissions from dung pats as a function of the identity and assemblage structure of dung beetles within them. The specific aims of this study were thus to test: *i)* whether different species displayed different GHG emission patterns; and, *ii)* whether mixed species performed differently from single-species assemblages.

## Materials and methods

To examine the functional consequences of variation in dung beetle assemblage composition, we established replicate laboratory terraria with four monospecific and two mixed assemblages, then recorded the outcome in terms of dung removal and on GHG emissions.

### Experimental design

**Fig 1:**
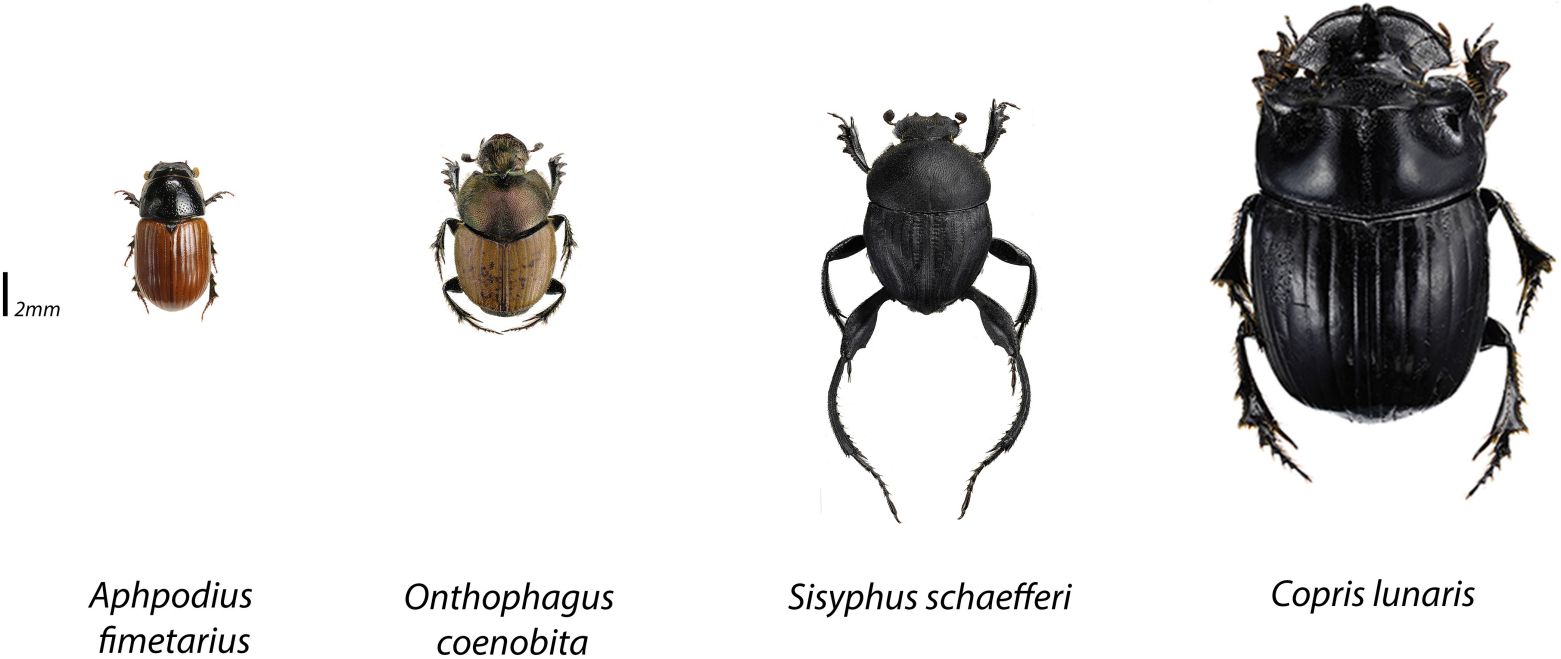
Species used in the experiment. The same pictograms are used to identify treatments in Figs 2–5. The length of each pictogram is proportional to the average body size of beetles, as adopted from [33]. Photographs by Göran Liljeberg.

Dung beetles were collected from La Mandria Natural Park (45° 08' 48.83'' N, 7° 36' 02.53'' E, 290 m above sea level), north-western Italy (using the same locality as [30]). This collection was authorized by the supervisory official of the “Ente di Gestione delle Aree Protette dei Parchi Reali” (Venaria, Italy). Species collected were neither endangered nor protected. Beetles were collected in May 2015, using standard cattle-dung-baited pitfall traps located in the broadleaf forest (dominated by *Quercus robur* and *Carpinus betulus*). Following [31], a total of 30 traps were interspersed by distances of at least 50 m, and the beetles collected after 48 hours. The design of our experiment was subsequently based on the snapshot of the local dung beetle fauna thus derived. Thus, the four species most abundantly caught were used in the experiment (Table 1): *Aphodius fimetarius* (Linnaeus, 1758) a small dweller; *Onthophagus coenobita* (Herbst, 1783), a small tunneler; *Sisyphus schaefferi* (Linnaeus, 1758), a small roller; and *Copris lunaris* (Linnaeus, 1758), a large tunneler (Fig 1) (with the classification into “small” and “large” species based on body mass, following [32]).

**Table 1:** Dung beetle species used in the experiment. The table identifies the nesting strategies, species, total number of individuals, mean individual dry body mass and number of individuals used in each experimental treatment.

Nesting strategies and beetle size		Species	Total number of individuals	Mean individual body mass [g]	Number of individuals in each treatment					
					Monospecific treatments				Mixed treatments	
					T1	T2	T3	T4	T5	T6
*Small dweller*		*Aphodius fimetarius*	413	0.01	31				17	11
*Tunneler*	*Small tunneler*	*Onthophagus coenobita*	161	0.02		13			6	4
	*Large tunneler*	*Copris lunaris*	14	0.20				2		
*Small roller*		*Sisyphus schaefferi*	56	0.05			6			2

To keep the total biomass of beetles at roughly 0.30 g per assemblage, species-specific numbers of individuals were varied between two and 31, with a minimum of two individuals per species (Table 1). This total biomass was chosen based on the mean total dung beetle biomass found in dung pats of 300g each in a previous pilot field study (mean value = 0.33g, SD. = 0.20g) (with more details in Appendix). Since the bigger species were of vastly larger size and biomass than the smaller ones, we chose to omit them from the mixed assemblages to maintain control over total biomass per treatment.

We ran six treatments (see Table 1): four monospecific treatments (T1-T4, each with one species only), and two mixed treatments (T5, T6) where the two versus three most abundant species, respectively, were included in proportions representative of field densities (Table 1). We also ran two controls: C1 dung without beetles, and C2 with neither dung nor beetles. Seven replicates were established for each treatment and control, thus yielding a total of 56 terraria (6 treatments x 7 replicates + 2 controls x 7 replicates = 56). We used terraria that consisted of a 16.5 litre plastic bucket (diameter 28 cm, height 27 cm). Since our experiment required a total of 576 litres of soil, we decided to use an artificial synthetic substrate rather than the natural soil from the site of origin. For this purpose, we used humus for gardening (0.5 Kg NPK 12-14-24 + 2MgO), homogenizing it through a 1cm-mesh. To reduce the organic content and to arrive at a composition similar to the natural substrate, we then mixed it with sand in a ratio of 1:2, following [34].

Fresh dung was collected from a herd of twelve Aberdeen Angus cattle grazing on natural grasslands dominated by graminoids (genera *Dactylis*, *Festuca*, *Poa*, *Lolium* and *Setaria*). No cow in the herd was treated with antibiotics or anthelmintics. The dung was manually homogenized before partitioning 300g to each treatment T1-T6 and to the control C1. This pat size was chosen from the range of typical pat sizes encountered in nature, selected to leave an uncovered strip of ground surrounding the pat.

The experiment lasted for 32 days, during which time the laboratory was kept at a constant temperature of 25° [35] and 60% humidity [36]. To simulate rain, we added 100 ml of deionized water to each terrarium after 8, 14, 19 and 24 days.

At the end of the experiment we weighed dry residual dung to evaluate the efficiency of dung removal for each treatment. By using dry weight, we controlled for any difference in evaporation, thereby isolating the contribution of the insects themselves in dung removal.

### Chamber and gas flux measurement

To evaluate gas fluxes from the terraria, we used a non-steady-state closed chamber technique [37,38] (overall approach adopted from [17], with additional details offered in Appendix). To close the terraria, we used lids organized with a vent tube and a sampling port following the USDA-ARS GRACEnet Chambers-base trace gas flux measurement protocol [39]. Between measurements, buckets were closed with a plastic mesh to avoid the escape of any dung beetles.

Gas fluxes were measured between 09:00am and 2:00pm on eight occasions between June 5^th^ and July 6^th^, with the timing of measurement (i.e. 1, 4, 7, 11, 15, 20, 26 and 32 days from the start of the experiment) following that of [17]. On each specific day, gas fluxes were measured in seven consecutive rounds, with each round encompassing one replicate of each treatment (T1, T2, T3, T4, T5, T6) and control (C1 and C2). The first round was initiated at 9:00 am, the last one at 1:30 pm. Gas fluxes measured during different rounds did not detectably differ from each other (see Table D in S1 Appendix).

Samples were taken with a syringe (50 ml) after 0, 8, and 16 minutes of the chamber being sealed, and injected into glass vials (12 ml). The contents of CO_2_, CH_4_ and N_2_O were then quantified in parts per million (ppm by volume) by a gas chromatograph (Agilent mod. 7890A) equipped with thermal conductivity, flame ionization and electron capture detectors.

Fluxes were calculated from the linear or nonlinear [40] increase over time (per hour) in concentration (selected according to the emission pattern) in the chamber headspace, as suggested by [38].

To evaluate the overall warming effect of GHG emissions from dung pats, compound-specific emissions should be combined. To weigh the fluxes together, we converted compound-specific fluxes of N_2_O and CH_4_ to CO_2_-equivalents by using the IPCC 2013 global warming potential (GWP) impact factors for 100 years’ time, i.e. 298 for N_2_O and 34 for CH_4_. These fluxes were then summed with the fluxes of CO_2_.

Following [17] and [20], cumulative fluxes of CO_2_, N_2_O, CH_4_, and CO_2_-equivalents were calculated for each chamber and expressed as areas under the curve (AUC) of the gas flux over time. For the period from the start of the experiment to day *i*, the cumulative area under the curve *A*_*i*_ was calculated as: ${Ai=\sum_{k=1}^{i}A}_{k}$. Accumulation rates (as trends in cumulative areas under the curves) and total cumulative fluxes (i.e. sums up to *i* = 32 days) were used as separate responses in subsequent analyses.

### Statistical analysis

Generalized Least Squares (GLS) models were used to analyse dung removal efficiency and gas fluxes, which allow the incorporation of autocorrelation structures (i.e. temporal dependencies between observations). To account for the heterogeneity of variance between treatments, we included a separate variance structure for each treatment where necessary. The most appropriate structure of random components was determined using a REstricted Maximum Likelihood (REML) estimation. The optimal random structure was determined by starting with a model without any variance–covariate terms (equivalent to linear regression) and comparing this model with subsequent GLS models that contained specific variance structures [41]. Comparisons of these models were made using Akaike Information Criteria (AIC) (Table B in S1 Appendix) and plots of residuals versus fitted values. Individual responses were modelled as follows.

To analyse how dung removal varied with dung beetle assemblage composition, residual dung weight was modelled as a function of treatment, while including a separate variance structure for each treatment.

To analyse how the fluxes (both hourly and cumulative) of different gases varied over time and treatments, a separate model was derived for each compound (CO_2_, CH_4_, N_2_O and CO_2_-equivalents, respectively). We run models that took into account the high variability within treatments and the temporal non-independence of consecutive measurements. Thus, each compound was modelled as function of measurement day and treatments, using terrarium as a random effect and including a separate variance structure for each treatment.

To analyse total cumulative fluxes at the end of the experiment, we applied a separate GLS to each compound, including a separate variance structure for each treatment. Consequently, each compound was modelled as function of treatments, including a separate variance structure for each treatment.

To adjust for multiple comparisons in all GLS models and post hoc test, we recalculated the p-value with a Holm correction (equal to a sequential Bonferroni correction; [42]). In other words, we multiplied the lowest p-value observed by the number (n) of independent tests conducted or by the number of independent variables, the next-lowest with n-1 etc. Both the adjusted and non-adjusted p-value are presented in the Tables in Appendix.

All analyses were performed using the ‘nlme’ package (v3.1–124; [43]) in the R (v3.2.1) statistical and programming environment (R Development Core Team 2005, for the R-scripts see Table A in S1 Appendix).

Data exploration of GHG fluxes per treatment and day highlighted the presence of an outlier from methane emissions of treatment T1 (day 3). This value was completely out of range of all other data, suggesting that it may come from an error during the gas extraction. For this reason, this data had not been taken into account in the analysis.

## Results

### Dung removal

**Fig 2:**
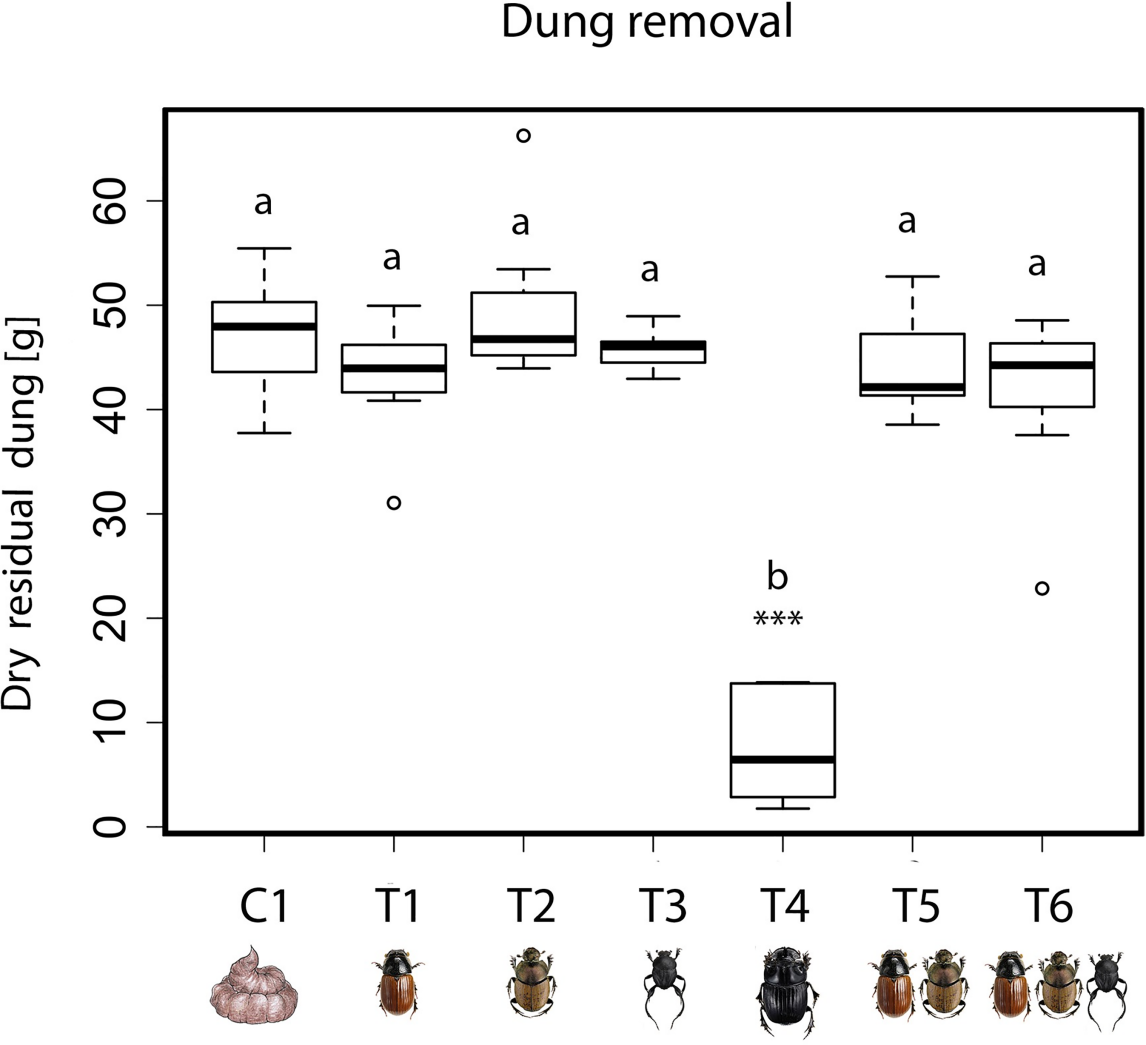
Dung removal in different treatments. Shown are box plots of the dry weight of dung (in grams) left at the end of the experiment. Letters above boxes identify differences among means as revealed by post-hoc analyses of GLS models. Boxes not sharing a letter were significantly different from each other, with significance levels derived from post-hoc analysis of the GLS model: ‘***’ = p<0.001.

At the end of the experiment the dry mass of dung remaining did not significantly differs between the treatments and the beetle-free control C1. The treatment T4 with *Copris lunaris* offered a notable exception, as this species removed much more dung than the others (Fig 2 and Table E in S1 Appendix).

### GHG emissions

**Fig 3:**
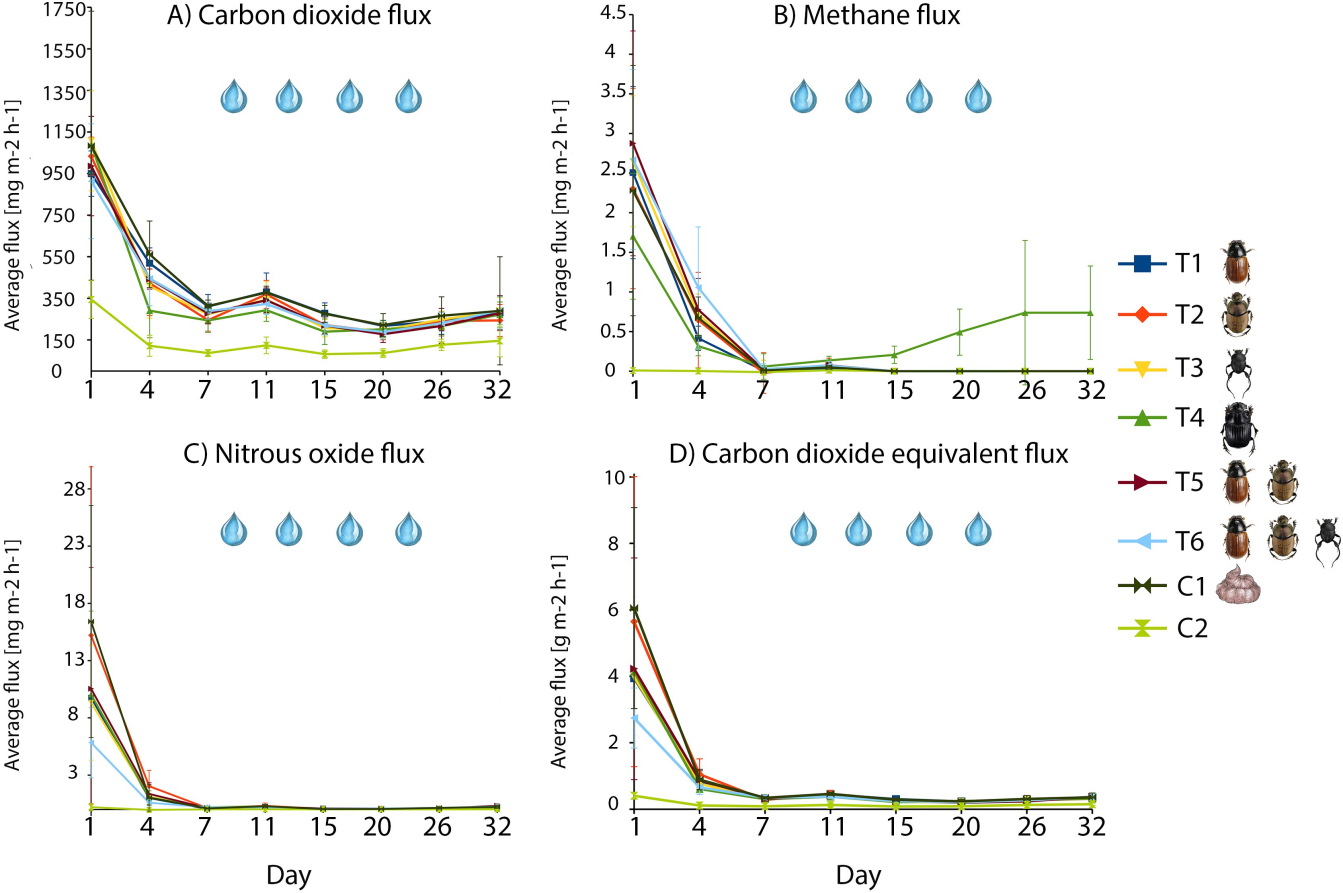
Compound-specific gas fluxes observed over time. Individual panels show fluxes of CO_2_ (a), CH_4_ (b), N_2_O (c) and CO_2_-equivalents (d), with each treatment represented by day-specific averages and standard deviations from empirical data. The water drops symbolizes the addition of 100 ml of deionized water to each terrarium. Error bars show standard deviations.

**Fig 4:**
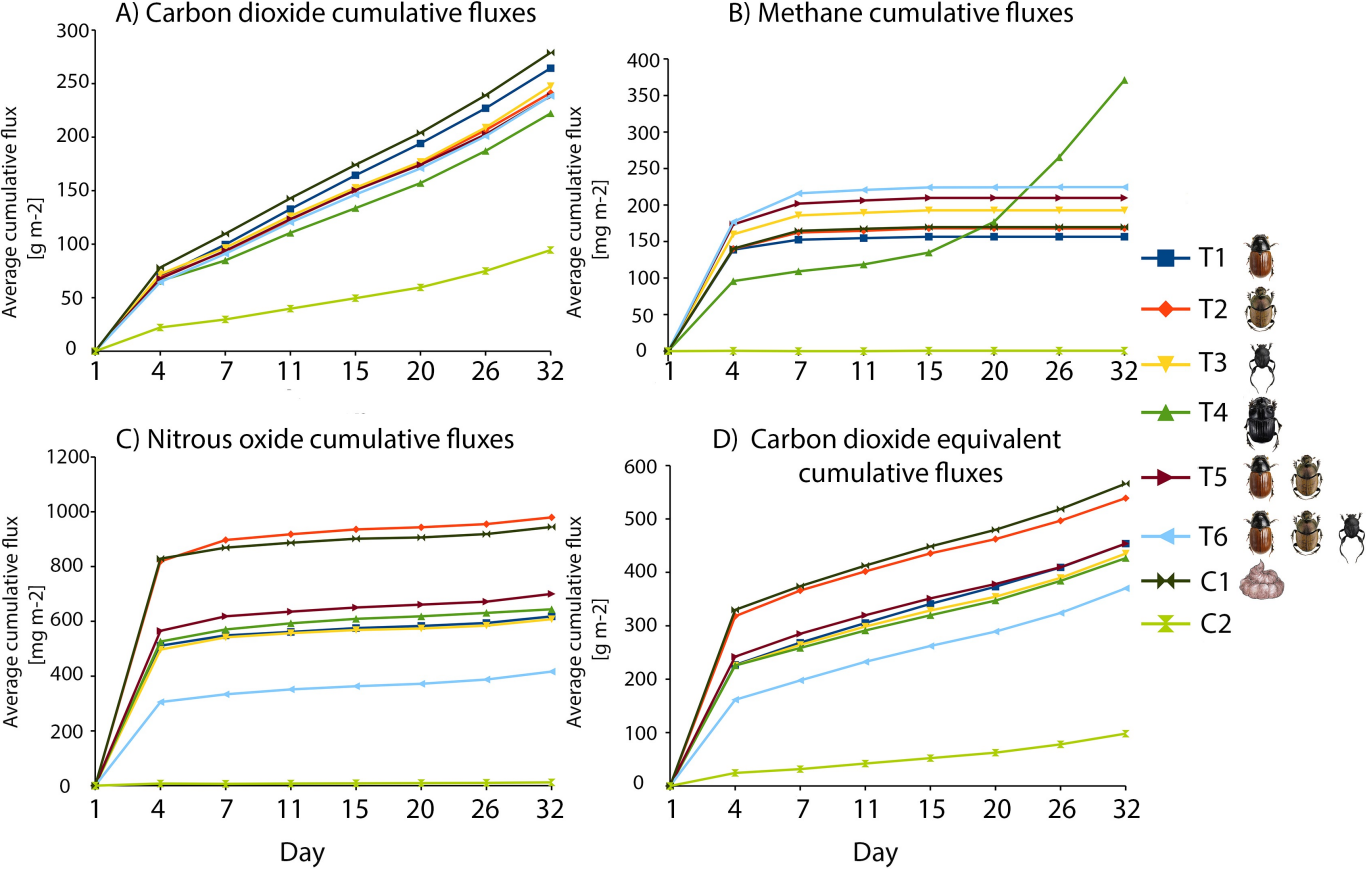
Compound-specific cumulative fluxes observed over time. Individual panels show cumulative fluxes of CO_2_ (a), CH_4_ (b), N_2_O (c) and CO_2_-equivalents (d) in different treatments and controls (see details and GLS result in Appendix).

**Fig 5:**
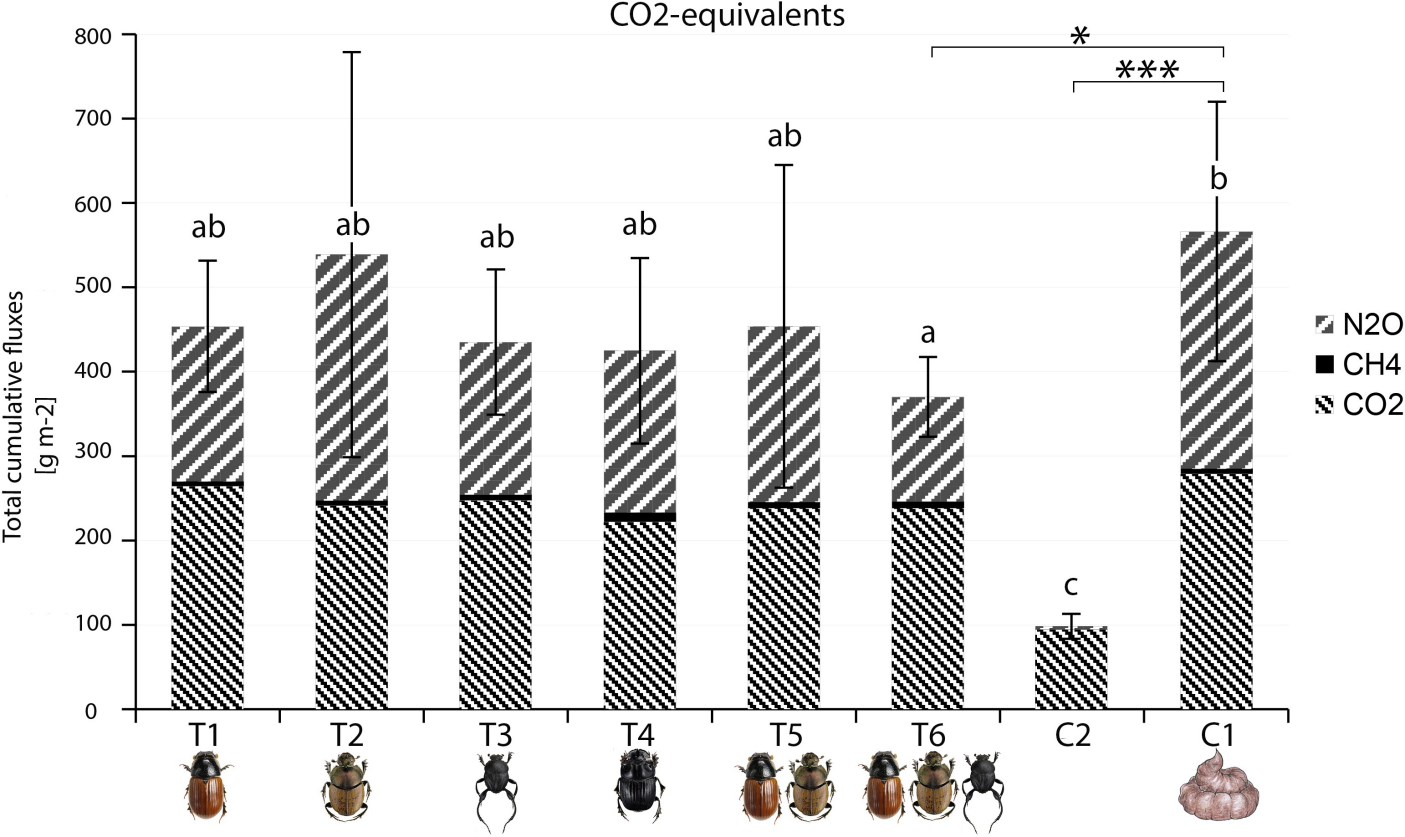
Total CO_2_-equivalents of greenhouse gases emitted in different treatments. To weigh together individual GHG compounds, we used compound-specific multipliers derived from IPCC (2013). Letters above bars identify differences among means revealed by post-hoc analyses of GLS models (more details in Table I in S1 Appendix). Bars not sharing the same letter were significantly different from each other. Stars define significant differences between terraria (treatment T6 and control C2 without dung) and reference category (control C1 with dung), revealed by GLS models. Error bars show standard deviations. ‘***’ = p<0.001 and ‘*’ = 0.05.

GHG fluxes from soil (i.e. from control C2, containing neither dung nor beetles) were much lower than fluxes from terraria with dung (Figs 3–5). Fluxes from dung pats decreased over time and showed different patterns among the compounds considered: while fluxes of all compounds were highest from fresh dung, this pattern was most pronounced for methane and nitrous oxide, which emissions essentially stopped within a week. By comparison, carbon dioxide fluxes continued–albeit at a reduced rate–throughout the duration of the experiment (Fig 3).

When focusing on the seven terraria types with dung pats (i.e. the set of six treatments with dung beetles and the control C1 containing dung only), the presence of dung beetles significantly reduced GHG emissions as compared to the control C1 containing dung only (Tables F and G in S1 Appendix).

#### Carbon dioxide

Emissions of CO_2_ varied between a maximum of 2421.15 mg C m^-2^ h^-1^ and a minimum of 23.96 mg C m^-2^ h^-1^ among the terraria with dung pats (treatments T1-T6 and C1; Fig 3A). CO_2_ fluxes differed among terraria (F_6,336_ = 2.57, p = 0.02, adjusted p = 0.057; with T1/T6 differing from C1) and time periods (F_7,336_ = 408.32, p<0.001, adj. p<0.001), with the size of the difference varying between time periods (Interaction treatments × days: F_42,336_ = 1.54, p = 0.02, adj. p = 0.04; for more details see Table F and G in S1 Appendix) (Figs 3A and 4A).

The cumulative CO_2_ flux was lower in all treatments than that of the control C1 (as containing dung only; T2: t_56_ = -2.25, p = 0.03, adjusted p = 0.12; T3: t_56_ = -1.91, p = 0.06, adjusted p = 0.12, T5: t_56_ = -2.50, p = 0.02, adjusted p = 0.08, T6: t_56_ = -2.18, p = 0.03, adjusted p = 0.12), but this difference was strong in the presence of *C*. *lunaris* (T4: t_56_ = -3.67, p<0.001, adj. p = 0.001; for more details see Table H in S1 Appendix) (Fig 4A).

#### Methane

Fluxes of CH_4_ ranged from a maximum of 5.73 mg C m^-2^ h^-1^ to a minimum of -0.55 mg C m^-2^ h^-1^ (treatments T1-T6 and control C1). For this compound, fluxes did not differ significantly among terraria (*F*_6,335_ *=* 1.03, p = 0.40, adj. p = 0.81), but only between time periods (*F*_7,335_ *=* 182.15, p<0.001, adj. p<0.001; interaction treatments × days: *F*_42,335_ *=* 1.58, p = 0.02, adj. p = 0.048; more details in Tables F and G in S1 Appendix) (Fig 3B). The same patterns were evident in cumulative CH_4_ fluxes (Fig 4B).

Total cumulative fluxes of CH_4_ from the beetle-free control C1 were significantly lower than those from the treatment with the big tunneler *C*. *lunaris* (treatment T4: t_*56*_ *=* 2.91, p = 0.00, adj. p = 0.037; more details in Table H in S1 Appendix). The emission pattern from this treatment (T4) changed over time, with CH_4_ emissions decreasing until the 7^th^ day, when they started to increase. As a result of this trend, cumulative emissions were lower than those from control C1 with dung at the beginning of the experimental period and higher at the end (Figs 3B and 4B).

#### Nitrous oxide

Fluxes of N_2_O varied between a maximum of 43.31 mg N m^-2^ h^-1^ and a minimum of -0.62 mg N m^-2^ h^-1^ among the terraria with dung pats (treatments T1-T6 and C1). The specific flux rates differed significantly among treatments over time (*F*_6,336_ *=* 2.27, p = 0.04, adj. p = 0.04) and over time (*F*_7,336_ *=* 95.64, p<0.001, adj. p <0.001; interaction treatment × days: *F*_42,336_ *=* 1.95, p<0.001, adj. P = 0.001; with more details in Tables F and G in S1 Appendix) (Figs 3C and 4C).

Cumulative N_2_O fluxes accumulated slower over time in treatments with beetles than in the control C1 with dung only (with the notable exception of treatment T2 containing the small tunneller, *Onthophagus coenobita*; Fig 4C). However, these differences were significant only between treatment T6 (with all three dung beetle species present) and the beetle-free control with dung, C1 (Treatment T6: t_*56*_ *=* -2.65, p = 0.01, adj. p = 0.07; more details in Table H in S1 Appendix).

#### CO_2_-equivalents

To the total fluxes of CO_2_-equivalents, CO_2_ and N_2_O contributed the majority, with a substantially smaller contribution from CH_4_ (Fig 5). Among the terraria with dung pats (treatments T1-T6 and control C1), emissions of CO_2_-equivalents differed significantly among terraria (*F*_6,336_ *= 2*.*68*, p = 0.02, adj. p = 0.02) and over time (*F*_7,336_ *=* 162.10, p<0.001, adj. p <0.001; interaction treatment × days: *F*_42,336_ *=* 2.14, p<0.001, adj. P = 0.001; with more details in Tables F and G in S1 Appendix) (Figs 3D and 4D).

Cumulative fluxes of CO_2_-equivalents accumulated slower in the presence (T1-T6) than in the absence (control C1) of beetles, with an average reduction of -21,33% (calculated from the data shown in Fig 5 as $\frac{T_{i}-C1}{C1}*100$; see [17]). The largest reduction was provided by the blend of three species (treatment T6), which was also significantly lower than that from the control C1 (Treatment T6: t_56_ = -3.22, p = 0.00, adj. p-value 0.02; more details in Table H in S1 Appendix; for post hoc analysis details in Table I in S1 Appendix) (Fig 5).

## Discussion

Where previous studies have revealed a general impact of dung beetles on GHG fluxes from cow pats [17,18,20], the current study reveals a new pattern: that the specific reduction in GHG emissions depends on the composition of the dung beetle assemblage. Quite surprisingly, we found the very same species to maximize the ecosystem service of dung removal and of carbon dioxide reduction and the ecosystem disservice of methane emissions from dung pats. These patterns come with two main implications: first, they support our *a priori* hypothesis that different dung beetle species, and different dung beetle assemblages, do indeed affect GHG fluxes differently. Second, they suggest that different ecosystem services may trade off against each other, and that functionally efficient organisms may simultaneously increase both desirable and undesirable ecosystem processes. Below, we will address each one in turn.

### Different dung beetle assemblages affect GHG emission differently

While previous studies have mainly targeted the overall effect of dung beetles on GHG emissions from dung pats [17,18], not all dung beetles are equal. Variation in nesting strategies [25] and in the body mass of species may significantly affect their functional efficiency [22,27,28,29,44]. Thus, we expected different beetles to affect GHG fluxes differently–a hypothesis for which we found direct support.

Even though our experimental design was explicitly based on the same total biomass of dung beetles in each experimental unit, assemblages of the large tunneler, *Copris lunaris*, released more total methane per unit beetle mass than did other beetle assemblages–and in fact, even more methane than did the control with dung only and no beetles. The exact patterns differed not only with the assemblage structure but with the GHG compound considered. When emissions of all compounds (CH_4_, N_2_O and CO_2_) were combined into the common currency of CO_2_-equivalents, dung beetle assemblages consisting of three species proved to release a full third (32%) less of GHGs than did beetle-free controls.

Exactly what processes are behind the patterns detected is yet to be clarified. For assemblages with *C*. *lunaris*, fluxes first decreased until day 7 of the experiment, then increased again. These patterns may reflect the nesting behaviour of this large tunneler, with decreasing CH_4_ fluxes during the first week corresponding to dung relocation into chambers before the brood ball formation starts [45]. During this period, *C*. *lunaris* manipulated and transported the dung into the soil, this may have enhanced its drying and increased the availability of oxygen. This may have decreased anaerobic decomposition and reduced methanogenesis (cf. [17]). Once in the larval chamber, brood balls will be kept moist by parental care, and may therefore continue to emit CH_4_ until the end of the experiment.

That the different activities of different dung beetle species may interact in determining the net functional outcome is suggested by the functional patterns emerging from monospecific versus mixed species assemblages.

Overall, the total emission of CO_2_ equivalents–i.e. the pooled climatic impact–was lower in the presence than in the absence of beetles and with the mix of three species providing the highest significant reduction. Yet, the exact mechanisms behind this desirable pattern of attenuation of GHG emissions in terms of CO_2_ equivalents are again to be targeted by further work. As our current experimental design was explicitly devised to resolve differences among species, and constrained by limitations on resources and overall terraria numbers, it falls short of resolving complementarity from facilitation effects (sensu [21])–or indeed any other specific mechanisms behind the patterns detected. Yet, it does suffice to generate the data-driven hypothesis that more diverse dung beetle communities may release less GHGs–an explicit hypothesis to be targeted by future experiments.

### Functionally efficient taxa may provide both ecosystem services and disservices

While the large tunneler *C*. *lunaris* was associated with unexpectedly high methane emissions, it was also the most efficient species in removing dung and reducing CO_2_ emissions, even more efficient than mixed assemblages. Thus, functionally efficient organisms may simultaneously increase both desirable and undesirable ecosystem processes [46,47] and different ecosystem services may trade off against each other [48,49]. Across different taxa, many species provide both ecosystem services and disservices. Important pollinators, as hawkmoth species (Lepidoptera: Sphingidae), have voracious herbivore larval-stages that, damaging the plants, have an effective fitness cost [50,51] and incur an indirect disservice for the crop. Ants provide several ecosystem services (reducing leaf herbivory, fruit pest damage and indirect pollination facilitation), but also disservices, increased mealybug density, phytopathogen dissemination and indirect pest damage enhancement [47]. Earthworms are also important as soil ecosystem engineers, they modify soil structure and interact with microbes through their feeding, burrowing and casting activities (ecosystem services) but it was proven that they also increase GHG emissions from soil (ecosystem disservice) [46].

Since dung beetles as a group are known to sustain a whole range of ecosystem services ranging from seed dispersal to parasite control [52], this points to a need for quantifying the relative role of species identity, functional diversity and overall species diversity for multiple functions at the same time. Dung removal *per se* seems not to reflect all other functions and services (current study; see also [53]), and a species excelling at producing one service may be either inefficient in producing another, or distressingly efficient in simultaneously sustaining a disservice.

## Conclusions

Overall, our study demonstrates that different dung beetle species contribute differently to dung removal and to GHG emissions from dung pats–and that one and the same species (*C*. *lunaris*) may contribute to both ecosystem services (dung removal and CO_2_ reduction) and disservices (increasing methane emissions). As different species may perform differently under different conditions [54], the best approach to safeguarding ecological functioning will be conserving diverse dung beetle communities [53].

In many countries worldwide, dung beetles are currently threatened by changes in pastoral practices and chemicalization of cattle farming [28,55–57]. As a case in point, the large tunneler, *C*. *lunaris*, which we identified as so functionally important in our study, has been declining in many parts of Northern Europe [33]. This and similar changes may incur unpredictable changes in the functioning of pasture ecosystems.

## Supporting information

S1 Appendix **Table A. Formula applied to each model.** Alternative models fitted to flux data, with the resultant AIC values offered in Table B. **Table B. AIC results for each model applied.** AIC values for each of the models outlined in Table A, as fitted to compound-specific gas fluxes. **Fig A. Terraria.** Buckets with lids organized with the vent port and the syringes for the gas extractions. **Table C. Respiration rates per mesocosm.** The respiration rate per each species was estimated using data available from [2]. To evaluate the beetle respiration per each mesocosm, the species respiration rate was multiplied by the number of individuals in each treatment. In order to compare the respiration rates with the data recorded in this experiment, the means of the CO_2_ fluxes recorded in the experiment were presented in the second column of the table. **Table D. Generalized Least Square models of GHG fluxes over measurement series** (i.e. gas fluxes were measured in different 7 rounds–series- from 9 to 13:30). Shown are estimates of GLS model of gas fluxes over time series with standard errors and statistical significance. Reference level: Series 1. Models were fitted assuming a Gaussian error distribution. **Table E. GLS models of dung removal.** Generalized least squares (GLS) models on residual dry dung (g) as a function of treatment. Shown are estimated coefficients with standard errors, t-value and statistical significance. Here, Control C1 was used as reference category. Column “p-value” refers to unadjusted probabilities derived from an t-distribution with the appropriate degrees of freedom, whereas column “Adjusted p-value” refers to probabilities after Holm-Bonferroni correction. For the latter, we multiplied the lowest p-value observed with the number (n) of independent variables, the next-lowest p-value with n-1 etc. (here: n *=* 7 independent variables). **Table F**. **GLS models of cumulative flux trends.** Generalized Least Squares models of the cumulative fluxes of CO_2_, CH_4_, N_2_O and CO_2_-equivalents among treatments (T1-T6) over time. Fluxes of CO_2_, CH_4_, N_2_O and CO_2_-equivalents, respectively, were modelled as a function of treatments and measurement time, i.e. days since the start of the experiment, used as a categorical variable. For further details, see Materials and methods. To estimate the specific effect of variation in the beetle assemblage on GHG emissions over time, we removed the control without dung (C2). Here, control C1 was used as reference category. Column “p-value” refers to unadjusted probabilities derived from an F-distribution with the appropriate degrees of freedom, whereas column “Adjusted p-value” refers to probabilities after Holm-Bonferroni correction. For the latter, we multiplied the lowest p-value observed with the number (n) of independent tests conducted, the next-lowest p-value with n-1 etc. (here: n *=* 4 separate compounds). **Table G**. **GLS models of hourly GHG flux over time.** Fluxes of CO_2_, CH_4_, N_2_O and CO_2_-equivalents, respectively, were modelled as a function of treatments and measurement time, i.e. days since the start of the experiment, used as a categorical variable. For further details, see Materials and methods. To estimate the specific effect of variation in the beetle assemblage on GHG emissions over time, we removed the control without dung (C2). Here, control C1 was used as reference category. Column “p-value” refers to unadjusted probabilities derived from an F-distribution with the appropriate degrees of freedom, whereas column “Adjusted p-value” refers to probabilities after Holm-Bonferroni correction. For the latter, we multiplied the lowest p-value observed with the number (n) of independent tests conducted, the next-lowest p-value with n-1 etc. (here: n *=* 4 separate compounds). **Table H. GLS models of cumulative GHG fluxes.** Total fluxes of CO_2_, CH_4_, N_2_O and CO_2_-equivalents, respectively, accumulated by the end of the experiment, were modelled as a function of treatment. The table shows compound-specific differences (columns) between treatments (as rows) control C2 (without beetles and dung) versus the control C1 (which include dung but no beetles) as reference category. Column “p-value” refers to unadjusted probabilities derived from an t-distribution with the appropriate degrees of freedom, whereas column “Adj. p-value” refers to probabilities after Holm-Bonferroni correction. For the latter, we multiplied the lowest p-value observed with the number (n) of independent variables, the next-lowest p-value with n-1 etc. (here: n *=* 8 independent variables). **Table I. Post hoc analysis of cumulative CO**_**2**_**-equivalents.** Cumulative emissions of CO_2_-equivalents, accumulated by the end of the experiment, were modelled as a function of treatment. Column “adjusted p-value” refers to probabilities after Holm-Bonferroni correction. For the latter, we multiplied the lowest p-value observed with the number (n) of independent variables, the next-lowest p-value with n-1 etc. (here: n *=* 28 total number of contrasts).
(PDF)
