# Supplementary material for: Greenhouse gas emissions from dung pats vary with dung beetle species and with assemblage composition
Source: PLoS One. 2017 Jul 12;12(7):e0178077. doi: 10.1371/journal.pone.0178077 (PMC5507485; doi:10.1371/journal.pone.0178077)
Supplement: S1 Appendix — Table A. Formula applied to each model. Alternative models fitted to flux data, with the resultant AIC values offered in Table B. Table B. AIC results for each model applied. AIC values for each of the models outlined in Table A, as fitted to compound-specific gas fluxes. Fig A. Terraria. Buckets with lids organized with the vent port and the syringes for the gas extractions. Table C. Respiration rates per mesocosm. The respiration rate per each species was estimated using data available from [2]. To evaluate the beetle respiration per each mesocosm, the species respiration rate was multiplied by the number of individuals in each treatment. In order to compare the respiration rates with the data recorded in this experiment, the means of the CO2 fluxes recorded in the experiment were presented in the second column of the table. Table D. Generalized Least Square models of GHG fluxes over measurement series (i.e. gas fluxes were measured in different 7 rounds–series- from 9 to 13:30). Shown are estimates of GLS model of gas fluxes over time series with standard errors and statistical significance. Reference level: Series 1. Models were fitted assuming a Gaussian error distribution. Table E. GLS models of dung removal. Generalized least squares (GLS) models on residual dry dung (g) as a function of treatment. Shown are estimated coefficients with standard errors, t-value and statistical significance. Here, Control C1 was used as reference category. Column “p-value” refers to unadjusted probabilities derived from an t-distribution with the appropriate degrees of freedom, whereas column “Adjusted p-value” refers to probabilities after Holm-Bonferroni correction. For the latter, we multiplied the lowest p-value observed with the number (n) of independent variables, the next-lowest p-value with n-1 etc. (here: n = 7 independent variables). Table F. GLS models of cumulative flux trends. Generalized Least Squares models of the cumulative fluxes of CO2, CH4, N2O and CO2-equiva [file pone.0178077.s001.pdf]

Appendix

1. Supplementary methods

Our experimental design was based on a constant biomass of 0.30 g among dung beetle assemblages (see main text). To determine the number of individuals to insert in the treatments, we weighted a minimum of ten dried individuals of each species with analytical balance (0.0001 mg), with the exact number weighted varying with the availability of specimens in the collection of University of Turin.

In order to establish the total biomass per each dung pat, we set up a pilot experiment in May 2015. We set up 10 300g-dung pats in a linear transect in an open pasture at IPLA (N45°05'20.9" E7°44'24.4"). Dung beetles were extracted after 48 hours of pat exposure. All species were counted and identified.

To evaluate gas fluxes from the dung pats, we used a closed chamber method. Once the lid was installed on the respective terrarium, the volume of the chamber without the dung pat was 3.077 litres. To avoid the stratification of the gases, we mixed the air inside the chamber (extracting it by syringe and re-expelling it again inside the chamber). The lid was gently replaced so as not to alter the pressure in the chamber.

Gas samples (35 ml) were drawn into 50 ml polypropylene syringes through a 2-way stopcock, 20 ml of gas was expelled to clean the needle and the remaining 30 ml gas was injected directly into 12-ml soda glass vials (Exetainer®, Labco Ltd., Buckinghamshire, UK). Each vial had been evacuated with a vacuum pump before use.

Gas fluxes were measured between 09:00am and 2:00pm on eight occasions between June 5th and July 6th. Specifically, the dates were 5th , 8th , 11th , 15th , 19th , 24th , and 30th June and the 6th July 2015, corresponding to days 1, 4, 7, 11, 15, 20, 26 and 32 of the experiment (following [1]).

Gas samples were taken after 0, 8, and 16 minutes of the chamber being sealed. The gas within the syringe was injected into a 12 ml vial.

In order to minimize sample contamination in case of leakage, and to allow multiple injections if needed, this procedure created an overpressure in the vial.

All three gases were analysed with gas chromatography, and the analysis were carried out within 5 days of extraction.

Instrument calibration was performed several times a day to avoid changes in atmospheric conditions during the analysis, with a three-point external calibration carried out with certified multi-standard gas samples (for CO_2, CH_4, N_2O; certified standard mixtures are provided by SIAD spa) at three different concentration levels.The calibration curve was recalculated after around 50 vials analysed. The nonlinearity of ECD response to N_2O concentration was corrected by a non-linear empirical function of the measured concentration.

The system was an Agilent mod. 7890A gas-chromatograph, equipped with a Gerstel Maestro MPS2 autosampler. After

the injection (injector temperature: 70°C) the sample was split into two lines for gas detection; line 1 was equipped with two packed columns (Supelco Sigma Aldrich Porapack Q and Porapack QS) kept at 80°C and with a TCD for CO_2 detection and a FID for methane detection, placed in-line. On this line it was used as carrier at 30.00 ml/min flow. Operating temperatures were 200°C for TCD and 250°C for FID. Line 2 was equipped with 2 packed columns (Sigma Aldrich Porapack Q) and with an ECD for N_2O detection; on this line a 5% Argon-Methane mix was used both as carrier and makeup (30.00 ml/min). ECD operating temperature was 350°C. All detectors were manufactured by Agilent Technologies. Each line was preceded by a 500 µL loop for sample volume determination; the system allowed sample edge and tail cutting by a two-valve system, in order to limit time analysis to nearly 6 minutes.

Minimum Detectable Concentrations for each gas were: 110 ppb for CH_4, 16.5 ppm for CO_2, 10 ppb for N_2O. MDF. Converted to fluxes (as based on MDC and chamber space), this corresponds to 0.02 mg m^{-2} h^{-1} for CH_4, 2.43 mg m^{-2} h^{-1} for CO_2, 0.0033 mg m^{-2} h^{-1} for N_2O. Fluxes that lay below detection limits were set to zero.

Table A: Formula applied to each model. Alternative models fitted to flux data, with the resultant AIC values offered in Table B.

Model	Formula	Correlation	Weights
GLMM	gas ~ Treatment*Day + (1|Terrarium).		
GAMM	gas ~ s(Day, k=5) + Treatment, random = list(Terrarium=~1), method = "REML".	Correlation=corAR1(0.8, form = ~ 1 | Treatment/Terrarium)	
LM	gas ~Treatment*Day , random=~1|Terrarium.	Correlation=corAR1(0.8, form = ~ 1 | Terrarium)	VarIdent(form= ~1|Treatment))
GLS	gas ~ Treatment*Day.	Correlation=corAR1(0.8, form = ~ 1 | Terrarium)	VarIdent(form= ~1|Treatment.

Table B: AIC results for each model applied. AIC values for each of the models outlined in Table A, as fitted to compound-specific gas fluxes.

	Model	AIC
CO_2	GLMM	568.4
	GAMM	786.3
	LME	490.6
	GLS	**491.8**
CH_4	GLMM	849.4
	GAMM	890.3
	LME	819.1
	GLS	**582.0**
N_2O	GLMM	959.1
	LME	648.6
	GAMM	976.6
	GLS	**646.6**
CO_2-equivalents	GLMM	836.4
	GAMM	872.1
	LME	570
	GLS	**570**

Figure A: Terraria. Buckets with lids organized with the vent port and the syringes for the gas extractions.

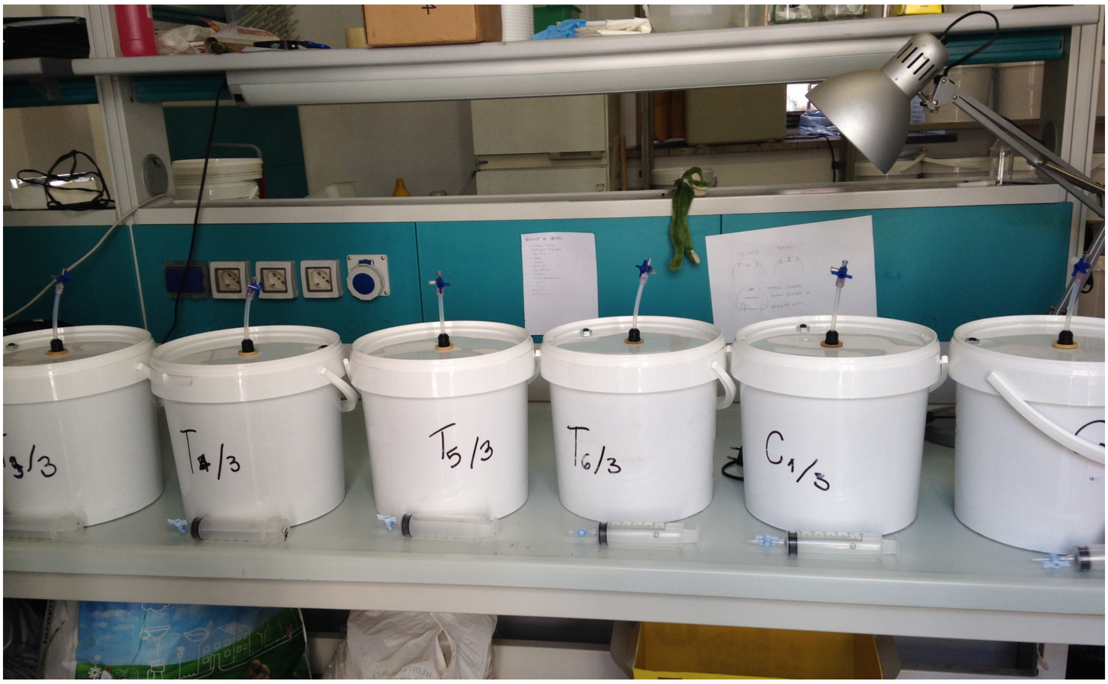

2. Supplementary results

Proportion of CO_2 fluxes emanating from beetle respiration

Part of the CO_2 fluxes observed in our terraria with beetles will have derived from respiration buy the beetles themselves. In order to have a rough estimation of this quantity, we derived a general relationship between dung beetle biomass and CO_2 emission, as based on data available from [2]. Our regression included the following species: *Sisyphus fasciculatus, Scarabaeus rusticus, Anachalcos convexus, Scarabeus flavicornis and Circellium bacchus*. More specifically, body mass accounted for 98% of variation in CO_2 emissions in respirometry system measurements (R^2=0.98, [2]). Applying the parameterised regression model to *Aphodius fimetarius*, *Onthophagus coenobita, Sisyphus schaefferi* and *Copris lunaris* suggests that a single individual of these species will emit: 0.000103, 0.000104, 0.000108 and 0.00012 g of CO_2 per hour. Converted to the number of individuals used per experiment, this suggests the assemblage-wide fluxes from the beetles themselves presented in Table C. Importantly, the estimates of [2] were derived for resting beetles, and respiration rates may be higher for active beetles (i.e. when flying, digging, etc). Nonetheless, realistic increases in respiration with activity will still not account for more than a fraction of the 30-fold difference in the beetle respiration versus total fluxes observed in the mesocosms (see Table C).

Table C: Respiration rates per mesocosm. The respiration rate per each species was estimated using data available from [2]. To evaluate the beetle respiration per each mesocosm, the species respiration rate was multiplied by the number of individuals in each treatment. In order to compare the respiration rates with the data recorded in this experiment, the means of the CO_2 fluxes recorded in the experiment were presented in the second column of the table.

Mesocoms	Beetle respiration per mesocosm (g/h)	Mean CO_2 fluxes (g/h) observed during the first day of the experiment
T1	0.00321	0.105
T2	0.00136	0.106
T3	0.00064	0.097
T4	0.0002483	0.096
T5	0.00238	0.101
T6	0.00177	0.111

Overall it is thus clear that respiration by the dung beetles themselves made an only weak contribution to overall CO_2 emissions observed.

Table D: Generalized Least Square models of GHG fluxes over measurement series (i.e. gas fluxes were measured in different 7 rounds – series- from 9 to 13:30). Shown are estimates of GLS model of gas fluxes over time series with standard errors and statistical significance. Reference level: Series 1. Models were fitted assuming a Gaussian error distribution.

GLS	**CO2**				**CH4**				**N2O**			
	Estimate	Std. Error	z value	p-value	Estimate	Std. Error	z value	p-value	Estimate	Std. Error	z value	p-value
Intercept	-0.14	0.12	-1.19	0.23	-0.39	0.01	-64.30	<0.001 ***	-0.31	0.01	-27.40	<0.001 ***
Series 2	0.08	0.17	0.45	0.65	-0.01	0.01	-1.33	0.18	-0.00	0.01	-0.02	0.98
Series 3	0.04	0.17	0.23	0.82	0.00	0.01	0.49	0.62	-0.00	0.01	-0.17	0.87
Series 4	0.15	0.17	0.91	0.36	0.00	0.01	0.26	0.79	-0.00	0.01	-0.00	0.99
Series 5	0.23	0.17	1.35	0.18	0.00	0.01	0.01	0.99	0.001	0.01	0.09	0.93
Series 6	0.09	0.17	0.55	0.58	0.01	0.01	1.01	0.31	-0.00	0.01	-0.03	0.98
Series 7	-0.06	0.17	-0.33	0.72	0.01	0.01	0.73	0.46	0.017	0.01	1.05	0.29

Table E: GLS models of dung removal. Generalized least squares (GLS) models on residual dry dung (g) as a function of treatment. Shown are estimated coefficients with standard errors, t-value and statistical significance. Here, Control C1 was used as reference category. Column "p-value" refers to unadjusted probabilities derived from an t-distribution with the appropriate degrees of freedom, whereas column "Adjusted p-value" refers to probabilities after Holm-Bonferroni correction. For the latter, we multiplied the lowest p-value observed with the number (n) of independent variables, the next-lowest p-value with n-1 etc. (here: n=7 independent variables).

	Dry residual dung				
	Estimate	Std. Error	t value	p-value	Adjusted p value
Int.	46.99	2.20	21.31	<0.001 ***	<0.001 ***
T1	-4.04	3.17	-1.28	0.21	0.83
T2	2.97	3.70	0.80	0.43	1
T3	-1.29	2.32	-0.55	0.58	1
T4	-39.10	3.08	-12.68	<0.001 ***	<0.001 ***
T5	-2.61	2.89	-0.90	0.37	1
T6	-5.73	4.01	-1.43	0.16	0.80

Table F: GLS models of cumulative flux trend. Generalized Least Squares models of the cumulative fluxes of CO_2, CH_4, N_2O and CO_2-equivalents among treatments (T1-T6) over time. Fluxes of CO_2, CH_4, N_2O and CO_2-equivalents, respectively, were modelled as a function of treatments and measurement time, i.e. days since the start of the experiment, used as a categorical variable. For further details, see Materials and methods. To estimate the specific effect of variation in the beetle assemblage on GHG emissions over time, we removed the control without dung (C2). Here, control C1 was used as reference category. Column "p-value" refers to unadjusted probabilities derived from an F-distribution with the appropriate degrees of freedom, whereas column "Adjusted p-value" refers to probabilities after Holm-Bonferroni correction. For the latter, we multiplied the lowest p-value observed with the number (n) of independent tests conducted, the next-lowest p-value with n-1 etc. (here: n=4 separate compounds).

Trend of cumulative flux over time					
Variables		Df	F value	p-value	Adjusted p-value
**CO_2**	Intercept	1	18.38	< 0.001 ***	< 0.001 ***
	Treatment	6	1.68	0.13	0.13
	Days	7	1709.19	< 0.001 ***	< 0.001 ***
	Treatment * Days	42	1.39	0.06	0.12
**CH_4**	Intercept	1	25.88	< 0.001 ***	< 0.001 ***
	Treatment	6	0.86	0.52	0.52
	Days	7	215.23	< 0.001 ***	< 0.001 ***
	Treatment * Days	42	2.71	< 0.001 ***	< 0.001 ***
**N_2O**	Intercept	1	74.12	< 0.001 ***	< 0.001 ***
	Treatment	6	1.63	0.14	0.14
	Days	7	120.54	< 0.001 ***	< 0.001 ***
	Treatment * Days	42	2.35	< 0.001 ***	< 0.001 ***
**CO_2-eq**	Intercept	1	94.78	< 0.001 ***	< 0.001 ***
	Treatment	6	2.09	< 0.001 ***	< 0.001 ***
	Days	7	469.43	< 0.001 ***	< 0.001 ***
	Treatment * Days	42	2.53	< 0.001 ***	< 0.001 ***

Table G: GLS models of hourly GHG fluxes over time. Fluxes of CO_2, CH_4, N_2O and CO_2-equivalents, respectively, were modelled as a function of treatments and measurement time, i.e. days since the start of the experiment, used as a categorical variable. For further details, see Materials and methods. To estimate the specific effect of variation in the beetle assemblage on GHG emissions over time, we removed the control without dung (C2). Here, control C1 was used as reference category. Column "p-value" refers to unadjusted probabilities derived from an F-distribution with the appropriate degrees of freedom, whereas column "Adjusted p-value" refers to probabilities after Holm-Bonferroni correction. For the latter, we multiplied the lowest p-value observed with the number (n) of independent tests conducted, the next-lowest p-value with n-1 etc. (here: n=4 separate compounds).

Analysis of hourly flux over time					
Variables	Df	F value	p-value	p-value	Adjusted p-value
**CO_2**	Intercept	1	0.91	0.34	0.34
	Treatment	6	2.57	0.02 *	0.057
	Days	7	408.32	< 0.001***	< 0.001***
	Treatment * Days	42	1.54	0.02 *	0.04 *
**CH_4**	Intercept	1	0.02	0.88	0.88
	Treatment	6	1.03	0.40	0.81
	Days	7	182.15	< 0.001 ***	< 0.001 ***
	Treatment * Days	42	1.58	0.02 *	0.048 *
**N_2O**	Intercept	1	14.13	< 0.001 ***	< 0.001 ***
	Treatment	6	2.27	0.04 *	0.04 *
	Days	7	95.64	< 0.001 ***	< 0.001 ***
	Treatment * Days	42	1.95	< 0.001 ***	< 0.001 ***
**CO_2-eq**	Intercept	1	14.61	< 0.001***	< 0.001***
	Treatment	6	2.68	0.02 *	0.02 *
	Days	7	162.10	< 0.001 ***	< 0.001 ***
	Treatment * Days	42	2.14	< 0.001 ***	< 0.001 ***

Table H: GLS models of cumulative GHG fluxes. Total fluxes of CO_2, CH_4, N_2O and CO_2-equivalents, respectively, accumulated by the end of the experiment, were modelled as a function of treatment. The table shows compound-specific differences (columns) between treatments (as rows) control C2 (without beetles and dung) versus the control C1 (which include dung but no beetles) as reference category. Column "p-value" refers to unadjusted probabilities derived from an t-distribution with the appropriate degrees of freedom, whereas column "Adj. p-value" refers to probabilities after Holm-Bonferroni correction. For the latter, we multiplied the lowest p-value observed with the number (n) of independent variables, the next-lowest p-value with n-1 etc. (here: n=8 independent variables).

Treat.	CO_2					CH_4					N_2O					CO_2-eq				
	Estimate	Std. Error	t value	p-value	Adj. p-value	Estimate	Std. Error	t value	p-value	Adj. p-value	Estimate	Std. Error	t value	p-value	Adj. p-value	Estimate	Std. Error	t value	p-value	Adj. p-value
Intercept	0.85	0.21	3.98	0.00 ***	0.00 ***	-0.13	0.26	-0.47	0.64	1	0.64	0.37	1.71	0.09	0.55	0.80	0.31	2.55	0.01 *	0.084
**C2**	-3.1	0.23	-13.2	0.00 ***	0.00 ***	-1.26	0.26	-4.77	0.00 ***	0.00 ***	-1.82	0.37	-4.86	0.00 ***	0.00 ***	-2.53	0.31	-8.02	0.00 ***	0.00 ***
**T1**	-0.24	0.28	-0.87	0.39	0.39	-0.10	0.33	-0.30	0.76	1	-0.64	0.44	-1.44	0.16	0.65	-0.61	0.35	-1.73	0.09	0.28
**T2**	-0.63	0.28	-2.25	0.03 *	0.12	-0.01	0.42	-0.03	0.97	1	0.07	0.72	0.09	0.92	0.92	-0.15	0.58	-0.25	0.80	0.80
**T3**	-0.53	0.27	-1.91	0.06	0.12	0.17	0.32	0.53	0.60	1	-0.66	0.43	-1.54	0.13	0.65	-0.71	0.36	-1.97	0.06	0.28
**T4**	-0.95	0.26	-3.67	0.00 ***	0.00 ***	1.50	0.52	2.91	0.00 **	0.037 *	-0.59	0.48	-1.23	0.23	0.68	-0.75	0.39	-1.95	0.06	0.28
**T5**	-0.68	0.27	-2.50	0.02 *	0.08	0.30	0.39	0.77	0.44	1	-0.48	0.57	-0.83	0.41	0.82	-0.61	0.50	-1.21	0.23	0.47
**T6**	-0.68	0.31	-2.18	0.03 *	0.1	0.41	0.43	0.95	0.35	1	-1.03	0.39	-2.65	0.01 *	0.07	-1.06	0.33	-3.22	0.00 **	0.02 *

Table I: Post hoc analysis of cumulative CO_2-equivalents. Cumulative emissions of CO_2-equivalents, accumulated by the end of the experiment, were modelled as a function of treatment. Column "Adjusted p-value" refers to probabilities after Holm-Bonferroni correction. For the latter, we multiplied the lowest p-value observed with the number (n) of independent variables, the next-lowest p-value with n-1 etc. (here: n=28 total number of contrasts).

Contrast	Estimate	SD	DF	t ratio	Adjusted p-value
C2 – C1	2.53	0.32	48	8.02	< 0.001 ***
C2 – T1	-1.92	0.16	48	-11.91	< 0.001 ***
C2 – T2	-2.38	0.49	48	-4.85	< 0.001 ***
C2 – T3	-1.82	0.18	48	-10.22	< 0.001 ***
C2 – T4	-1.78	0.23	48	-7.82	< 0.001 ***
C2 – T5	-1.92	0.39	48	-4.91	< 0.001 ***
C2 – T6	-1.47	0.10	48	-14.51	< 0.001 ***
C1 – T6	1.06	0.33	48	3.22	0.048

Reference

1. Penttilä A, Slade EM, Simojoki A, Riutta T, Minkkinen K, Roslin T. Quantifying beetle-mediated effects on gas fluxes from dung pats. PLoS One. 2013; 8: e71454.

2. Duncan F D, Byrne M. J. Discontinuous gas exchange in dung beetles: patterns and ecological implications. Oecol. 2000; 122(4), 452-458.
